# Retraction Note: Oral supplementation of diabetic mice with propolis restores the proliferation capacity and chemotaxis of B and T lymphocytes towards CCL21 and CXCL12 by modulating the lipid profile, the pro-inflammatory cytokine levels and oxidative stress

**DOI:** 10.1186/s12865-023-00594-9

**Published:** 2023-12-20

**Authors:** Ahmad A. Al Ghamdi, Gamal Badr, Wael N. Hozzein, Ahmed Allam, Noori S. Al-Waili, Mohammed A. Al-Wadaan, Olivier Garraud

**Affiliations:** 1 Chair of Engineer Abdullah Baqshan for Bee Research, College of Food and Agriculture Sciences, King Saud University, Riyadh, Saudi Arabia; 2 Laboratory of Immunology and Molecular Physiology, Zoology Department, Faculty of Science, Assiut University, Assiut, 71516 Egypt; 3 Bioproducts Research Chair, Department of Zoology, College of Science, King Saud University, Riyadh, Saudi Arabia; 4 Botany Department, Faculty of Science, Beni-Suef University, Beni-Suef, Egypt; 5 Zoology Department, Faculty of Science, Beni-Suef University, Beni-Suef, Egypt; 6 Al-Waili’s Foundation for Science, New York, USA; 7 Institut National de la Transfusion Sanguine, Paris, Université de Lyon, Saint-Etienne, France

**Retraction Note: BMC Immunology (2015) 16:54**

The Editor has retracted this article. After publication, concerns were raised regarding the flow cytometry data presented in the Figures. Specifically:

- Irregular flow cytometry plot quality in Fig. 3a and b;
- Highly similar features between the right sides of the plots in Fig. 3b;
- Unusual appearance and highly similar features between different plots in Fig. 4a.

The authors have stated that the original data are no longer available. Due to the number and severity of the issues with the data, the Editor no longer has confidence in this article.

Gamal Badr does not agree to this retraction. None of the other authors have responded to any correspondence from the editor or publisher about this retraction.

